# The Anti-Inflammatory Effects of Vitamin D in Tumorigenesis

**DOI:** 10.3390/ijms19092736

**Published:** 2018-09-13

**Authors:** Wei Liu, Lei Zhang, Hui-Jing Xu, Yan Li, Chuan-Min Hu, Jing-Yan Yang, Mei-Yan Sun

**Affiliations:** 1 College of Laboratory Medicine, Jilin Medical University, Jilin 132013, China; liuweijldx@sina.com (W.L.); zhanglei0358@sina.com (L.Z.); renyunqingzheng@126.com (H.-J.X.); jlliyang2004@163.com (Y.L.); 2 Laboratory of Antibody Engineering, Jilin Medical University, Jilin 132013, China; chuminhu@163.com; 3 Department of Physical Education, Jilin Medical University, Jilin 132013, China; yjy8899@126.com

**Keywords:** vitamin D, inflammation, tumorigenesis, cytokines, immune cells

## Abstract

In conjunction with the classical functions of regulating intestinal, bone, and kidney calcium and phosphorus absorption, as well as bone mineralization of vitamin D, the population-based association between low vitamin D status and increased cancer risk is now generally accepted. Inflammation is causally related to oncogenesis. It is widely thought that vitamin D plays an important role in the modulation of the inflammation system by regulating the production of inflammatory cytokines and immune cells, which are crucial for the pathogenesis of many immune-related diseases. Mechanistic studies have shown that vitamin D influences inflammatory processes involved in cancer progression, including cytokines, prostaglandins, MAP kinase phosphatase 5 (MKP5), the nuclear factor kappa B (NF-κB) pathway, and immune cells. Multiple studies have shown that vitamin D has the potential to inhibit tumor development by interfering with the inflammation system. The present review summarizes recent studies of the mechanisms of vitamin D on regulating the inflammation system, which contributes to its potential for cancer prevention and therapy. This review helps answer whether inflammation mediates a causal relationship between vitamin D and tumorigenesis.

## 1. Introduction

Vitamin D is a fat-soluble steroid derivative, which plays an important role in calcium homeostasis and bone metabolism through its actions in intestine, bone, kidney, and the parathyroid glands [1,2,3]. Moreover, preclinical and clinical studies strongly suggest that vitamin D deficiency increases the risk of developing multiple malignancies. Inflammation is appropriately added as one of the 10 hallmarks of cancer [4]. Cytokines and immune cells in the inflammatory microenvironment essentially serve as direct growth and migratory factors for cancer cells [5,6]. Studies have shown that tissues with chronic inflammation generally exhibit high cancer incidence [7,8,9]. Vitamin D can modulate the innate and adaptive immune responses. Studies of tumor cells revealed that vitamin D exerts important regulatory effects on some of the key molecular pathways involved in inflammation. Studies have shown the role of vitamin D in the inflammatory microenvironment, but the evidence linking vitamin D and immune response in the context of cancer is still scarce. In the current review, we aim to provide an overview of anti-inflammatory actions and vitamin D in tumorigenesis; the results suggest the beneficial effects of vitamin D supplementation in decreasing the risk and adverse outcomes of cancer, although the precise effect remains to be elucidated in large clinical trials.

## 2. Vitamin D and Epidemiology

Epidemiological data showed that vitamin D deficiency is significantly associated with high risk of multiple tumors and poor prognosis [10,11,12,13]. Garland et al. first determined that low levels of calcitriol may be associated with high incidence of colorectal cancer [14]. This hypothesis was subsequently verified in a large number of meta-analyses, including breast, prostate, colon, lung, and other cancers [15]. The results showed that high serum 25(OH)D levels are negatively correlated with the incidence or mortality of multiple cancers [16]. A meta-analysis of 17,332 cancer patients indicated that a 10 nmol/L increase in the blood 25(OH)D level confers a 4% reduction in overall mortality of cancer patients and high vitamin D levels are significantly associated with tumor prognostic indicators [17]. In a large case-cohort study within a Japan Public Health Center-based prospective study, the results showed that higher vitamin D concentrations are associated with lower risk of total cancer. These findings support the hypothesis that vitamin D has protective effects against cancer [18].

The correlation between vitamin D and the risk of gastrointestinal cancer were mostly reported. Gorham et al. found that daily intake of vitamin D above 1000 IU reduces the risk of colorectal cancer by about 50% for people who receive less than 100 IU daily vitamin D intake [19]. Ma et al. found that high-dose vitamin D intake and high levels of serum 25(OH)D reduce the risk of colorectal cancer by 12% and 33%, respectively [20]. The vitamin D intake of the respondents was estimated by the Health Professionals Follow-Up Study and the Nurses’ Health Study based on 120,000 men and women, including 365 confirmed cases of pancreatic cancer, during a 16-year follow-up. The results of the study showed that respondents who consumed more vitamin D in their diet had a lower incidence of pancreatic cancer than those with a lower vitamin D intake [21]. Biochemical evidence clearly indicated that hepatic carcinoma cells are responsive to the inhibitory effect of vitamin D. Severe 25(OH)D deficiency identifies a poor prognosis in patients with hepatocellular carcinoma [22].

Vitamin D has also been implicated in the development and progression of other cancers [23]. Several studies suggested that decreased serum levels of 25(OH)D are correlated with increased risk of prostate cancer (PCa) [24]. Studies have shown that, for a 20 ng/mL increase in serum 25(OH)D levels, the risk of breast cancer is reduced by 26%, suggesting that the risk of breast cancer is significantly reduced with the increase in serum vitamin D levels [25]. Kim et al. performed a meta-analysis of vitamin D intake, serum 25(OH)D concentrations, and the incidence and prognosis of breast cancer. A dose-response analysis of 13 studies showed that every 100 IU/day increase in vitamin D intake decreases the incidence of breast cancer by 2% [26].

## 3. Vitamin D and Metabolism

**Figure 1:**
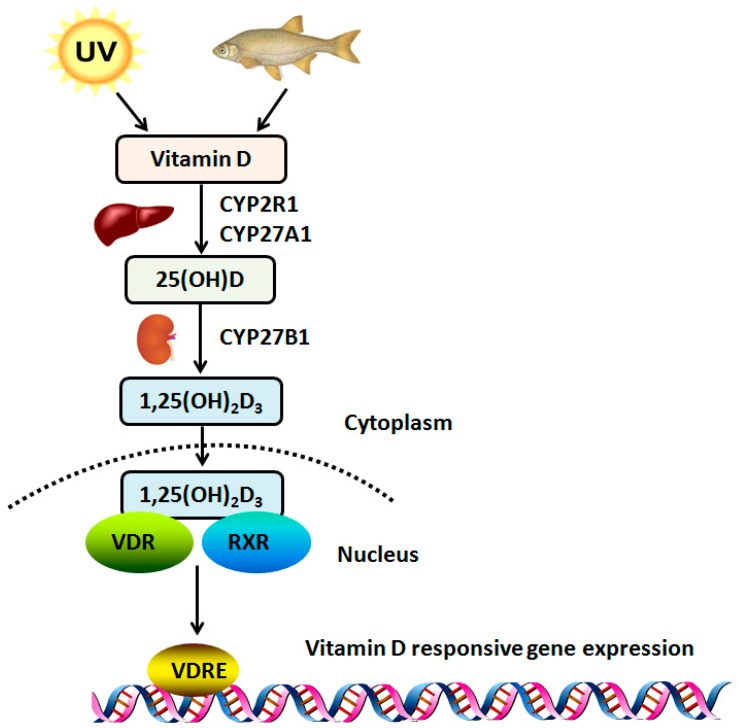
Vitamin metabolism. Vitamin D is synthesized by UVB radiation in the epidermis of skin or obtained from diet, hydroxylated to form 25(OH)D by CYP2R1 and CYP27A1 in liver, further metabolized by CYP27B1 in kidney to 1,25(OH)2D3 (calcitriol), and binds to the vitamin D receptor (VDR) which then heterodimerizes with retinoid X receptor (RXR). The 1,25(OH)_2_D_3_-VDR-RXR complex binds to promoter regions (VDREs) of vitamin D-responsive genes to modulate gene expression.

Vitamin D3 (D_3_) is the main form of vitamin D in the human body, which can be either synthesized from 7-dehydrocholesterol when skin is exposed to ultraviolet-B (UVB) light or obtained from the diet (Figure 1). In the liver, vitamin D is metabolized by vitamin D 25-hydroxylase (*Cytochrome P450 2R1*, *CYP2R1*) and sterol 27-hydroxylase (*Cytochrome P450 27A1*, *CYP27A1*) to 25(OH)D, which is the major circulating form of vitamin D in serum. 25(OH)D is further metabolized by 25(OH)D 1α-hydroxylase (*Cytochrome P450 27R1*, *CYP27B1*) in the mitochondria of the kidney epithelial cells to 1,25-dihydroxy vitamin D3 (1,25(OH)_2_D_3_), also known as calcitriol [27]. In some cancers, such as parathyroid carcinomas, the expression levels and activity of *CYP27B1* in the cancer cells are lower than in normal cells [28]. Hsu et al. compared CYP27B1 activity in samples from normal prostate epithelial cells, cancer-derived prostate epithelial cells, PCa cells lines, and samples of benign prostatic hyperplasia. *CYP27B1* expression is significantly reduced in the benign prostatic hyperplasia cells and is reduced further in the cancer-derived cells and cell lines. Decreased expression of *CYP27B1* is correlated with a decrease in growth inhibition in response to 25(OH)D [29]. In order to maintain the homeostasis of the organism, especially regarding the levels of calcium and phosphate, the enzyme 1,25 dihydroxyvitamin D3 24 hydroxylase (*Cytochrome P450 24R1*, *CYP24R1*) plays a key role in converting calcitriol to biologically inactive metabolites [30]. Hobaus et al. found that 77 (60%) out of 127 colorectal tumors show increased *CYP24A1* gene copy-number and that more than six copies of *CYP24A1* are positively correlated with *CYP24A1* mRNA expression suggestive of a causal relationship [31]. Albertson et al. proved that the amplification of the *CYP24A1* gene in human breast tumors and analysis of the datasets from The Cancer Genome Atlas confirms that a subset of human breast cancers (10–13%) exhibit alterations in the *CYP24* gene, with the most frequent changes being amplifications and up-regulation at the mRNA level [32]. Borkowski et al. found that high *CYP24A1* expression is significantly correlated with poor patient outcome in multiple lung cancer cohorts [33]. Better understanding the regulation of vitamin D hydroxylases in tumorigenesis may provide targeting strategies in the future.

Calcitriol attaches to a member of the ligand-activated transcription factor steroid hormone receptor superfamily, known as the VDR, which displays a typical domain structure of a nuclear receptor with a highly conserved DNA-binding region and ligand-binding domain. Binding between the ligand-binding domain and calcitriol (or its analogs) induces heterodimerization of VDR and the RXR to form the calcitriol-VDR-RXR complex, binding the complex to vitamin D response elements (VDREs) in multiple regulatory regions, located at promoters and distal sites of target genes, which, along with the recruitment of co-modulators, plays an important role in regulating cell proliferation and differentiation [34,35]. The presence of the VDR in tumor cells is a prerequisite for the antineoplastic effects of calcitriol. The cell growth evaluated by MTT assay is greatly increased in CYP24-induced and VDR-diminished cells than non-responding cells by 25(OH)D_3_ activity (*p* < 0.01). In addition, 23 cases of low *VDR* expression have a poorer prognosis than 19 cases of high *VDR* expression [36]. Lopes et al. investigated the immunohistochemical expression of the *VDR* in situ in a range of benign lesions and carcinomas of the mammary gland. The percentage of positive cases for the VDR is higher in benign lesions than in invasive tumors (93.5% and 56.2%, respectively) [37].

## 4. Vitamin D and Inflammation

**Figure 2:**
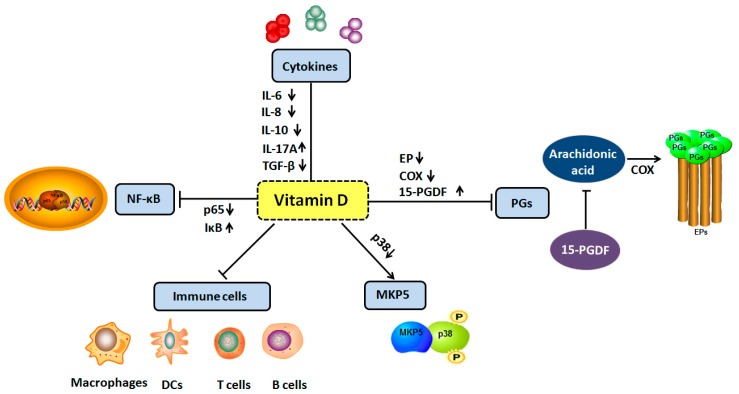
The effects of vitamin D of inflammation in tumorigenesis. These include the following. (1) Regulate the levels of cytokines including *IL-6*, *IL-8*, *IL-17A*, *IL-10 and TGF-β*; (2) inhibit NF-κB signaling pathway; (3) up-regulate the expression of MAP kinase phosphatase 5; (4) inhibit the prostaglandins pathway via reducing PG receptors (EPs), decreasing *COX-2* expression and increasing *15-PGDH* expression; (5) inhibit the immune cells via VDR including macrophages, DCs, B cells and T cells. Upward arrows indicate activation, downward arrows indicate inhibition.

In the context of cancer, the role of the immune system is not straightforward. Interactions can occur between cancer cells and host immune cells in the tumor microenvironment to create an immunosuppressive network that promotes tumor growth and protects the tumor from immune attack [38]. Population-based studies, as well as molecular studies, have demonstrated that vitamin D is implicated in many immune-related diseases, such as asthma, atherosclerosis, type 2 diabetes, and autoimmune diseases [39,40]. Recent studies indicated that a persistent inflammatory microenvironment can induce tumor production [41,42,43,44,45,46]. In the course of the development of the tumor, some immune cells often change from the “protector” of self-organizations to the “accomplice” of tumor cells, and nourish the cancer [47]. By mediating complex pathways, the tumor inflammatory microenvironment induces the expression of a variety of pro-inflammatory cytokines, promotes angiogenesis and tumor growth, invasion and metastasis, and accelerates the development of tumors [48,49,50,51]. Several mechanisms for how vitamin D affects inflammatory microenvironment in cancers have been explored, including regulating the interaction between immune and tumor cells to regulate the levels of cytokines, inhibiting NF-κB signaling pathway, up-regulating MKP5, and inhibiting the prostaglandins pathway and immune cells (macrophages, DCs, B cells, and T cells) [52,53] (Figure 2). Non-steroidal anti-inflammatory drugs (NSAIDs) are generally used to relieve acute pain and treat chronic inflammation, such as arthritis, among which the most commonly used are aspirin, ibuprofen, and piroxicam. A recent study has confirmed that NSAIDs have a certain degree of anti-tumor effect, which further confirms the close relationship between inflammation and cancer [54].

### 4.1. Vitamin D and Cytokines

Cytokines produced in the inflammatory microenvironment have been correlated with cancer pathogenesis. Several cytokines are found to be higher in the blood of colorectal cancer patients than in healthy controls [55]. By microarray-based methods, Powell et al. found that several cytokine genes (*IL-1β*, *IL-6*, *IL-8*, *and TGF-β1*) are over-expressed in PCa patients compared to European American men [56]. Interestingly, studies revealed that vitamin D exerts critical regulatory effects on some cytokines’ effects on natural and acquired immunity. Barrat et al. found that vitamin D leads to enhanced *IL-10* gene expression and inhibition of the *Th1*- and *Th2*-specific transcription factors [57]. Dauletbaev et al. demonstrated that high concentrations of 25(OH)D_3_, 1,25(OH)_2_D_3_ and the synthetic analogue paricalcitol moderately down-regulate *IL-8* in hyperinflammatory macrophages from cystic fibrosis patients [58].

IL-6 is a pro-inflammatory cytokine with pro-tumorigenic capacity and is a crucial key effector in PCa and colorectal cancer progression [59]. For example, IL-6 up-regulates *Mcl-1* gene transcription through p38-MAPK and JAK-STAT pathways, inhibiting tumor necrosis factor-relatedapoptosis inducing ligand (TRAIL)-induced tumor cells apoptosis [60]. The results showed that IL-6, as the effect factor of COX-2, can promote angiogenesis and tumor growth [61]. Cathcart et al. demonstrated that IL-6 down-regulates p53 protein levels and results in a concomitant increase in *MMP-14* expression, leading to enhanced cancer cell invasion and metastasis [62]. Several reports have shown that vitamin D may influence the regulation of IL-6 synthesis. 25(OH)D pretreatment inhibits both UV- and tumor necrosis factor alpha (TNF-α)-stimulated IL-6 production in normal cells via p38 MAPK inhibition, demonstrating its significant anti-inflammatory effects in cancer cells [63].

IL-8 has been found to be an important angiogenic factor. It can promote the initiation, migration, and invasion of tumors by promoting the proliferation of neoplastic cells and expression of *MMP-2* and *MMP-9*, increasing collagenase activity and inhibiting tumor cell apoptosis [64,65]. In the study by Singh et al., IL-8 activity was observed to play a crucial role in breast cancer, being regulated by HER-2 positive cancers through chemokine receptor 1/2 (CXCR1/2) ligands. The *IL-8* levels influence breast cancer stem cell activity by enhancing or down-regulating tumorigenesis [66]. Bao et. al found that calcitriol affects the stability of *IL-8* mRNA through the ATTTA motif in the 3′-flankingregion or other post-transcriptional regulations, thereby delaying PCa cell-induced human umbilical vein endothelial cell migration and tube formation [67]. Another study in colon cancer cells showed that calcitriol reduces the production of IL-8, and the anti-inflammatory effect is associated with depression of IL-8 production due to increased release of the soluble form CD14 (sCD14) depending on ERK1/2 [68].

IL-10 is an important anti-inflammatory cytokine due to its effects in down-regulating *Th1* cytokine (*IL-2* and *IFN-γ*) and *Th2* cytokine (*TNF-α*, *IL-6*, and *IL-8*) production, and its potential to promote resolution of inflammation [69,70]. *IL-10* normally plays an important role in controlling the development of chronic inflammatory processes and its absence results in chronic inflammatory responses (particularly in the gut), which are probably induced by normal enteric antigens [71]. IL-10 could inhibit the production of pro-inflammatory cytokines, which in turn stimulates tumor growth [72]. In a mouse model of colitis anti-CTLA-4, treatment induces IL-10^+^ Tregs that express the cell surface receptor inducible co-stimulator ligand (ICOS) with potent indoleamine 2,3 dioxygenase (IDO)-dependent anti-inflammatory properties [73]. These observations are particularly interesting as expression of *ICOS* is an immunologic marker correlated with clinical responses in PCa patients, who receive anti-CTLA-4 therapy [74]. It has been observed that the expression of *TLR9* by human adaptive IL-10-Treg populations is regulated by calcitriol [75,76].

TGF-β promotes tumor invasiveness through MMP-2, MMP-9, MT-MMP1 and urokinase-like plasminogen activator is up-regulated in both pancreatic ductal adenocarcinoma and hepatocarcinoma [77]. TGF-β1 induces integrin *αvβ3* expression and the enhanced expression of *αvβ3* potentiates TGF-β1-induced responses, which enhance epithelial-mesenchymal transition (EMT) in mammary epithelial cells in lung fibroblasts [78]. Artaza et al. found that vitamin D suppress TGF-β-mediated fibrosis through modulating multiple pro-fibrotic proteins, for instance, lowering collagen I and III expression and raising expression levels of *MMP-8* in mesenchymal multipotent cells [79].

### 4.2. Vitamin D and Prostaglandins

Prostaglandins (PGs) are pro-inflammatory molecules that bind to specific receptors and play a key role in mediating a series of cellular activities, such as cell proliferation, differentiation, and apoptosis, and promote tumorigenesis and cancer growth [80,81,82,83,84]. PG-related metabolic enzymes and EPs in the inflammatory microenvironment suggests that PGs are closely related to the occurrence and development of these tumors [85].

PGs mediate different signal transduction pathways through the interaction with EPs [86]. More importantly, the interactions between prostaglandin E2 (PGE2) and EP2 can produce positive feedback signals to increase TNF-α, IL-6, CXCL1, and COX-2 levels of neutrophils and tumor-associated fibroblasts [87], thereby promoting tumorigenesis by amplifying inflammation and shaping the inflammatory microenvironment [88,89]. Bazzani et al. indicated PGE2 induces epidermal growth factor receptor (EGFR) nuclear translocation and growth through the release of EGFR ligands in lung adenocarcinoma cells [90]. Moreno et al. further confirmed the mechanisms of suppressing the biological activity of PGs by vitamin D. Calcitriol decreases the mRNA expression of the *PGE2*, inhibits the oncogene *COX-2*, and induces the expression of the putative tumor suppressor *15-PGDH* in prostate cells, suggesting that calcitriol may play an important role in the chemoprevention of PCa [91].

COX-2 is a key enzyme in the synthesis of PGs. Clinical trials and epidemiological studies have suggested that COX-2 is involved in tumorigenesis and its inhibition can reduce the risk of cancer [92,93,94]. Studies have shown that COX-2 can up-regulate the expression of vascular endothelial growth factor, such as *VEGF-D* (lymphangiogenic factor) and *VEGFR-3* (VEGF-D receptor), which promote tumor angiogenesis in inflammatory mammary carcinomas [95]. High expression of *COX-2* in lung adenocarcinoma cells has also been found to activate tyrosine kinase receptor activity and induce over-expression of the *VEGF-C* gene, which in turn promotes tumor angiogenesis [96]. *COX-2* over-expression in tumor cells also stimulates production of VEGF-A, which causes blood vascular endothelial cell migration and tube formation [97]. On the other hand, over-expression of *COX-2* has a significant inhibitory effect on the apoptosis of tumor cells. Recent studies have shown that COX-2 can inhibit the apoptosis of tumor cells by activating the *Bcl2* gene pathway. The selective COX-2 inhibitor NS-398 can significantly inhibit the function of tumor cells to secrete PGE2 and maintain the cells in G1 phase [98,99]. In PCa cells, calcitriol reduces the expression levels of *COX-2* [100]. Moreno et al. found that calcitriol abolishes c-fos induction and growth stimulation by arachidonic acid, which reflects the effect of calcitriol in decreasing endogenous synthesis of PGs due to COX-2 suppression in PCa cells [91]. In addition, calcitriol interferes with the COX-2/PGE2 pathway, inhibits the activity levels of the phosphorylation of *ERK* and *ERα*, then downregulates the expression of *CYP1B1*, consequently inhibiting the proliferation of breast cancer cells [101].

15-hydroxyprostaglandin dehydrogenase (15-PGDH) is an antagonist of COX-2 and exerts a strong inhibitory effect on the development of cancer [102,103]. Yan et al. suggested that the TGF-β-mediated induction of 15-PGDH and crosstalk between the TGF-β and prostaglandin pathways may represent a significant additional effector of TGF-β-mediated suppression of cancer in the gastrointestinal tract [104]. Studies have shown that the absence or reduction of *15-PGDH* promotes the development of tumors in breast cancer, medullary thyroid carcinoma, PCa, and bladder cancer [105,106,107,108], thus supporting a tumor-suppressor role for 15-PGDH in cancer. Several studies highlighted the calcitriol-mediated suppression of the oncogene *COX-2*, an increase in the expression of the putative tumor suppressor *15-PGDH* in prostate cells, a decrease in the levels of biologically active PGs, thereby reducing the development of cancer [91]. The up-regulation of the *IGFBP-3* gene has been shown to be crucial in calcitriol-mediated inhibition of LNCaP cell growth. Calcitriol regulation of androgen-responsive genes, as well as genes involved in androgen catabolism, suggests that there are interactions between calcitriol and androgen signaling pathways in LNCaP cells [109].

In conclusion, vitamin D inhibits the synthesis and biological actions of pro-inflammatory PGs by three mechanisms: reducing PG receptors, decreasing *COX-2* expression, and increasing *15-PGDH* expression.

### 4.3. Vitamin D and MAP Kinase Phosphatase 5

MKP5 is another novel calcitriol-responsive gene. MKP5 preferentially binds and inhibits the activation of p38 MAPK, which is a family of serine/threonine-directed kinases classified as stress-activated kinases. A consequence of p38 activation is an increase in the production of pro-inflammatory cytokines that sustain and amplify the inflammatory response [110,111]. Calcitriol up-regulates *MKP5* expression, leading to subsequent inhibition of the production of pro-inflammatory cytokines, such as *IL-6*, by interfering with the signaling of pleiotropic inflammatory cytokines, such as *TNFα*, supporting a role for calcitriol in the prevention and/or early treatment of PCa [63].

### 4.4. Vitamin D and Nuclear Factor Kappa B Signal Pathway

NF-κB transcription factor is a heterodimer consisting of p50 and p65. It binds to its inhibitory protein inhibitor of NF-κB (IκB) and exists as an inactive hetero-oligomer in the cytoplasm. NF-κB is vital for the regulation of genes that control various responses in eukaryotic cells [112]. Studies revealed that calcitriol inhibits inflammatory responses by modulating the NF-κB signal pathway. Calcitriol inhibits lipopolysaccharide (LPS)-induced p38 phosphorylation and TNF-α and IL-6 production through increased binding of the VDR and histone H4 acetylation at the identified VDRS of the murine and human MKP1 promoters [63,113]. Calcitriol can also increase IκB levels by increasing mRNA stability and decreasing its phosphorylation, thereby reducing the nuclear translocation of NF-κB [114]. Calcitriol suppresses activation of the p65 subunit of the NF-κB complex in colon cancer cells, thereby preventing binding of NF-κB to DNA [115,116]. Thus, calcitriol may serve as an effective inhibitor for cancer via suppression of the NF-κB signal pathway.

### 4.5. Vitamin D and Immune Cells

As the VDR is expressed on immune cells (B cells, T cells, and antigen presenting cells, such as macrophages and DCs), vitamin D could modulate the innate and adaptive immune response [117]. There is increasing epidemiologic evidence linking vitamin D deficiency and autoimmune diseases, including multiple sclerosis (MS), rheumatoid arthritis (RA), diabetes mellitus (DM), inflammatory bowel disease, and systemic lupus erythematosus (SLE) [118,119,120]. At present, the role of vitamin D in reducing inflammation-dependent carcinogenesis via regulating immune cells is not straight forward, but it is generally recognized that the interactions occurring between tumor and host immune cells in the tumor microenvironment could promote tumor growth and protect the tumor from immune attack. We addressed several mechanisms, through which vitamin D affects different immune cells.

The relationships between B cells and vitamin D have been less intensively studied, but current research suggests that vitamin D can be used as a modulator of allergic immune responses by affecting B cells. Chen et al. found that it inhibits the ongoing proliferation of activated B cells and induces their apoptosis by up-regulating the expression of *p27*, whereas initial cell division is unimpeded. These results indicate that calcitriol may play an important role in the maintenance of B cell homeostasis [121]. Vitamin D deficiency impairs rituximab-mediated cellular cytotoxicity and the outcome of patients with diffuse large B cells lymphoma [122].

A large variety of immune cells express the VDR. Notably, expression of *VDR* increases on T cells upon antigenic stimulation. Jeffery et al. observed that stimulation of CD4^+^ CD25^−^ T cells in the presence of calcitriol inhibits production of pro-inflammatory cytokines, including IFN-γ, IL-17, and IL-21, induced high levels of *CTLA-4* and *FoxP3*, but does not substantially affect T cell division [123]. T-cell cytokines also control vitamin D metabolism in macrophages. For example, IFN-γ, a Th1 cytokine, up-regulates the macrophage CYP27B1, leading to enhanced bioconversion of 25(OH)D_3_ to its active metabolite-calcitriol. In contrast, the Th2 cytokine IL-4 induces catabolism of 25(OH)D_3_ to the inactive metabolite 24,25(OH)_2_D_3_ [124]. Sheikh et al. observed that the stimulation of CD4^+^ T cells with vitamin D suppresses proliferation capacity; enhanced the expression of *PD1*, *PD-L1*, and *CTLA-4* inhibitory markers on CD4^+^ T cells; and diminished the percentage of pro-inflammatory cytokines, including IFN-γ, IL-17, and IL-22, except IL-4 in CD4^+^ T cells [125]. Conditional targeting experiments showed that VDR function in T cells is necessary. Neither calcitriol nor T-cell-specific VDR targeting influences CD4^+^ Foxp3^+^ T-cell proportions in the periphery or the central nervous system (CNS) [126]. Palmer et al. suggested that vitamin D deficiency may promote autoimmunity by favoring the inordinate production of Th17 and Th9 cells at the expense of regulatory IL-10-producing T cells [127]. Calcitriol suppresses the inflammatory infiltrates and inhibits the expression of *P65*, *RORγt*, and *IL-17* in the spleen tissues of model mice [128]. These results suggest a potential mechanism, through which vitamin D metabolism links the T-cell-mediated immune responses to the adaptive immune responses.

The innate immune system interacts with vitamin D in several interesting ways. First, the primary role of macrophages is to engulf and kill bacteria. Toll-like receptors (TLRs) activation of human macrophages up-regulates expression of the *VDR* and the vitamin *CYP27B1* genes, leading to induction of the antimicrobial peptide cathelicidin and killing of intracellular *Mycobacterium tuberculosis* [129]. Chen et al. found a novel regulatory mechanism for vitamin D to control innate immunity: calcitriol promotes negative feedback regulation of TLR signaling via targeting microRNA-155-SOCS1 in macrophages [130]. Moreover, calcitriol or its analogs have been shown to initiate the differentiation of myeloid progenitors into macrophages, and to reduce *MCP-1* and *IL-6* expression via inhibiting the activation of NF-κB in macrophages [131]. Helming et al. found that calcitriol can selectively suppress the key effector functions of IFN-γ-activated macrophages. The deactivation of IFN-γ-stimulated macrophages is dependent on a functional VDR and calcitriol acts specifically on IFN-γ-activated macrophages, whereas the steroid has no effects on resting macrophages [132].

Dendritic cells (DCs) are the most potent antigen-presenting cells. DCs modulate T cell development and they can be not only immunogenic but also tolerogenic, both intrathymically and in the periphery [133]. A number of studies demonstrated that calcitriol inhibits the differentiation, maturation, and immunostimulatory capacity of human DCs. Studies have consistently reported that in vitro treatment of DCs with VDR agonists leads to down-regulated expression of the costimulatory molecules *CD40*, *CD80*, and *CD86*, and to decreased *IL-12* and enhanced *IL-10* production, resulting in decreased T cells activation [134,135]. Szeles et al. found that monocyte-derived DCs are able to turn on calcitriol sensitive genes in the early phases of differentiation if the precursor is present. Their data collectively suggest that exogenously- or endogenously-generated calcitriol autonomously regulates a large set of its targets not via inhibition of differentiation and maturation, leading to the previously characterized tolerogenic state [136]. Takeda et al. observed a significant increase in Foxp3 (+) regulatory T cells and a decrease in CD80^+^CD86^+^DCs in the mesenteric lymph nodes, spleen, and atherosclerotic lesions in oral calcitriol-treated mice in association with increased *IL-10* and decreased *IL-12* mRNA expression. CD11c^+^ DCs from the calcitriol group showed reduce proliferative activity of T lymphocytes, suggesting the suppression of DC maturation [137]. Cytokines secreted by vitamin D-treated DCs are significantly more potent in driving differentiation of IL-22-producing T cells, as compared to secreted cytokines of not-vitamin D-treated DCs [138].

## 5. Conclusions

A large number of epidemiological surveys have shown that vitamin D deficiency is related to the high incidence of many types of tumors. Experimental data have also confirmed that vitamin D can inhibit the occurrence and metastasis of tumors through many different processes. Among the above, factors related to the inflammatory microenvironment are attracting considerable attention. The treatment of tumor inflammatory microenvironment components as a new target can overcome the limitations of many current traditional treatment methods. Although multiple molecular pathways of vitamin D action in cancer cells have been identified, these pathways provide a mechanistic basis for its potential efficacy via anti-inflammatory actions in cancer. The mechanism of vitamin D in inhibiting tumors by affecting the tumor microenvironment needs to be further explored and studied. Vitamin D and its analogues as anti-cancer drug candidates still need additional prospective intervention experiments to provide direct evidence.
